# An antibody scanning method for the detection of α-synuclein oligomers in the serum of Parkinson's disease patients†

**DOI:** 10.1039/d2sc00066k

**Published:** 2022-09-22

**Authors:** Klara Kulenkampff, Derya Emin, Roxine Staats, Yu P. Zhang, Laila Sakhnini, Antonina Kouli, Oded Rimon, Evgeniia Lobanova, Caroline H. Williams-Gray, Francesco A. Aprile, Pietro Sormanni, David Klenerman, Michele Vendruscolo

**Affiliations:** Centre for Misfolding Diseases, Yusuf Hamied Department of Chemistry, University of Cambridge Cambridge CB2 1EW UK mv245@cam.ac.uk; Yusuf Hamied Department of Chemistry, University of Cambridge Cambridge CB2 1EW UK dk10012@cam.ac.uk; UK Dementia Research Institute, University of Cambridge Cambridge CB2 0XY UK; John Van Geest Centre for Brain Repair, Department of Clinical Neurosciences, University of Cambridge UK

## Abstract

Misfolded α-synuclein oligomers are closely implicated in the pathology of Parkinson's disease and related synucleinopathies. The elusive nature of these aberrant assemblies makes it challenging to develop quantitative methods to detect them and modify their behavior. Existing detection methods use antibodies to bind α-synuclein aggregates in biofluids, although it remains challenging to raise antibodies against α-synuclein oligomers. To address this problem, we used an antibody scanning approach in which we designed a panel of 9 single-domain epitope-specific antibodies against α-synuclein. We screened these antibodies for their ability to inhibit the aggregation process of α-synuclein, finding that they affected the generation of α-synuclein oligomers to different extents. We then used these antibodies to investigate the size distribution and morphology of soluble α-synuclein aggregates in serum and cerebrospinal fluid samples from Parkinson's disease patients. Our results indicate that the approach that we present offers a promising route for the development of antibodies to characterize soluble α-synuclein aggregates in biofluids.

## Abstract 2

Misfolded α-synuclein oligomers are closely implicated in the pathology of Parkinson's disease and related synucleinopathies.

## Graphical abstract

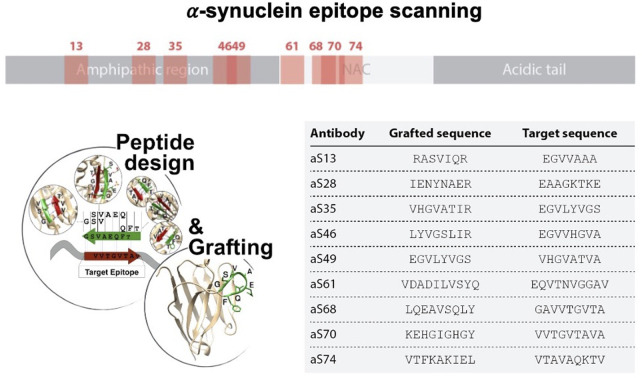

## Introduction

The aggregation of α-synuclein is a hallmark of a family of neurodegenerative diseases collectively known as synucleinopathies, the most common one being Parkinson's disease (PD),^1^ which is estimated to affect over 6 million people worldwide.^2^ PD is characterized by the presence of Lewy bodies in the midbrain of affected individuals, which are proteinaceous deposits that contain α-synuclein aggregates.^3^ Its close association with PD has made α-synuclein a central target for therapeutic and diagnostic interventions.^4–6^ As the pathology of other misfolding diseases, soluble prefibrillar aggregates, known as oligomers, rather than mature aggregates, have been identified as the driver of cytotoxicity.^7–10^ However, not much is currently known about the structure and abundance of α-synuclein oligomers in normal and diseased brains.^11,12^

In recent studies α-synuclein oligomers have been detected in the blood and cerebrospinal fluid (CSF) of PD patients,^13–16^ opening the way for the potential use of these particles as biomarkers both for diagnostics and surrogate endpoints in clinical trials.^12^ Methods to detect these aggregates^12,17^ include immunoassays such as ELISA^18–20^ or single-molecule immunoassays,^21–23^ which rely on the use of highly specific antibody pairs.^9–11^ Commonly available antibodies used in these immunoassays target either linear epitopes at the highly immunogenic C-terminal region of α-synuclein, including phosphorylated forms, or aggregate conformations of this protein (*e.g.* mouse monoclonal antibodies targeting residues 121–125 of α-synuclein (SC),^18,19^ α-synuclein phosphorylated at Ser129 (PS-129),^20,23^ or oligomeric α-synuclein (Syn-O2)^20^). However, the development of conformation-specific antibodies against pathogenic α-synuclein assemblies, including oligomers, remains challenging.^24^ It has also proven difficult to target regions outside the C-terminus because of their low immunogenicity, in particular the central non-amyloid-β component (NAC), which plays an important role in the aggregation process and formation of the intermediate aggregate species.

A recently introduced method of rational design of antibodies offers an opportunity to scan protein sequences to address this problem.^25,26^ In previous studies, a battery of antibodies was generated using this approach against the amyloid β peptide (Aβ), and subsequently grafted onto a single-domain antibody scaffold.^27^ After expression and purification, the designed antibodies (DesAbs) were characterized in different assays.^27^ By selecting DesAbs with low affinity for monomeric and fibrillar forms, a conformation-specific antibody to Aβ oligomers was identified.^28^ It was also shown that, as different DesAbs bind different Aβ epitopes, they can be used to characterize different Aβ aggregates with different mechanisms of toxicity.^29,30^

In this study, we applied this antibody design method to rationally design a panel of antibodies scanning the sequence of α-synuclein. Characterizing the binding of each DesAb in various biophysical assays including a kinetic aggregation assay and by fluorescence microscopy allowed us to identify a DesAb capable of recognizing α-synuclein oligomers in the serum of PD patients. By using super-resolution microscopy, we estimated the size distribution of these oligomers, consistent with recent findings that these species are conformationally heterogeneous.^31^

## Results and discussion

### Generation of a library of DesAbs to scan the sequence of α-synuclein

**Fig. 1:**
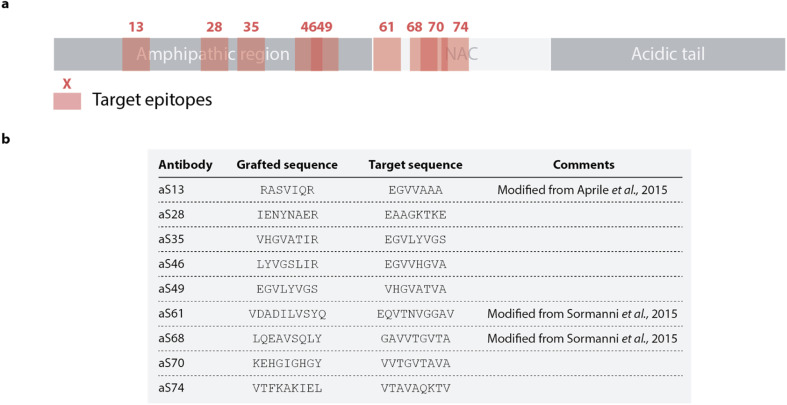
Rational design of a panel of 9 DesAbs against α-synuclein. (a) Schematic representation of the 9 targeted epitopes (pink) along the α-synuclein sequence. (b) List of antibody designs and target sequences. Antibodies are named after the position of the target epitope on α-synuclein.

In this study, we sought to apply a rational antibody design approach recently used to identify an oligomer specific antibody against Aβ to scan the sequence of α-synuclein. Nine designed peptides to be used as paratopes targeting selected epitopes in the amphipathic and NAC regions of α-synuclein were thus selected. This approach produced, for each target epitope, complementary peptides (paratopes) by combining sequence fragments found to form β-sheet pairs with fragments of the target epitope in protein structures available in the Protein Data Bank (PDB).^25,26^ To rank complementary peptides, each sequence is given a score comprising factors such as the frequency the β-sheet pairs are found in the PDB or the specificity for each binding pair.^25,26^ The solubility for the binding partners is evaluated by employing a sequence-based solubility calculation with the CamSol method^32^ and DesAbs scoring a high solubility score are favored. The designed complementary peptides and the target sequence are shown in Fig. 1 with the position of the epitope labelled with regards to the sequence of α-synuclein. The fragments forming each peptide design are shown in Fig. S1.† Each DesAb was expressed and purified (Fig. S2†), correct folding (Fig. S3†) and thermal stability (Fig. S4†) were verified by circular dichroism.

### Different DesAbs have distinct effects on the aggregation of α-synuclein

We measured the activity of the nine DesAbs described above in a kinetic aggregation assay of α-synuclein under mildly acidic and quiescent conditions.^33^ Under these conditions, α-synuclein undergoes autocatalytic nucleation on existing fibril surfaces, rather than elongation of fibrils by incorporating monomeric protein on to fibril ends. Aggregation reactions were initiated by the addition of a fixed concentration of preformed fibrils. The formation of fibrils was then tracked by a fluorescent readout of the amyloid-specific dye thioflavin T (ThT). Performing the aggregation assay in the presence of varying concentrations of antibodies and fitting all the data simultaneously (global fit) enables the assessment of the effects of the antibodies on the microscopic steps in the aggregation of α-synuclein.^33,34^

**Fig. 2:**
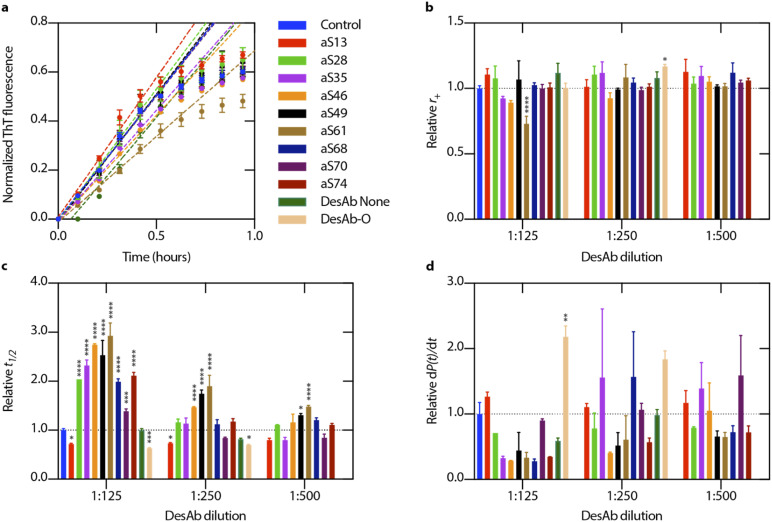
Designed antibodies show different effects on microscopic rates in α-synuclein aggregation. (a) Designed antibodies (DesAbs) were incubated in the presence of α-synuclein supplemented with a high concentration of preformed α-synuclein seeds (15% of monomer concentration). Error bars represent ±s.d. of three replicates. To determine the elongation rate, *r*_+_ = 2*k*_+_*m*_tot_, the first six data points were fitted with a linear function (eqn (1)). (b) A plot of the extracted elongation rates for various antibody concentrations is shown in comparison to the control reaction. (c) Relative half-time values for each antibody and concentration that were assessed, expressed as *t*_1/2_. (d) For each antibody and concentration observed, relative fibril amplification rates, d*P*(*t*)/d*t* (eqn (6)), are shown as an estimate for secondary nucleation, *r*_2_ = *k*_2_*m*_tot_^*n*_2_^. Error bars represent ±s.d. of three technical replicates. Data were analysed using a one-way analysis of variance (ANOVA) and all *p*-values are given compared to the mean of the control result (no antibody present in aggregation reaction). *p < 0.1, **p < 0.01, ***p < 0.001, ****p < 0.0001.

All DesAbs were first tested in a high seeded assay (15% of monomeric α-synuclein concentration) to decouple the nucleation and elongation mechanisms. Under high seed conditions, the effect of primary and secondary nucleation can be neglected, thus enabling the quantification of the effects on fibril elongation. Each of the DesAbs which was incubated at varying concentrations in the highly-seeded assay had an insignificant effect on the aggregation and the elongation rate, *k*_*+*_, as quantified by fitting the initial, linearly increasing data points with a linear function (Fig. 2).

**Fig. 3:**
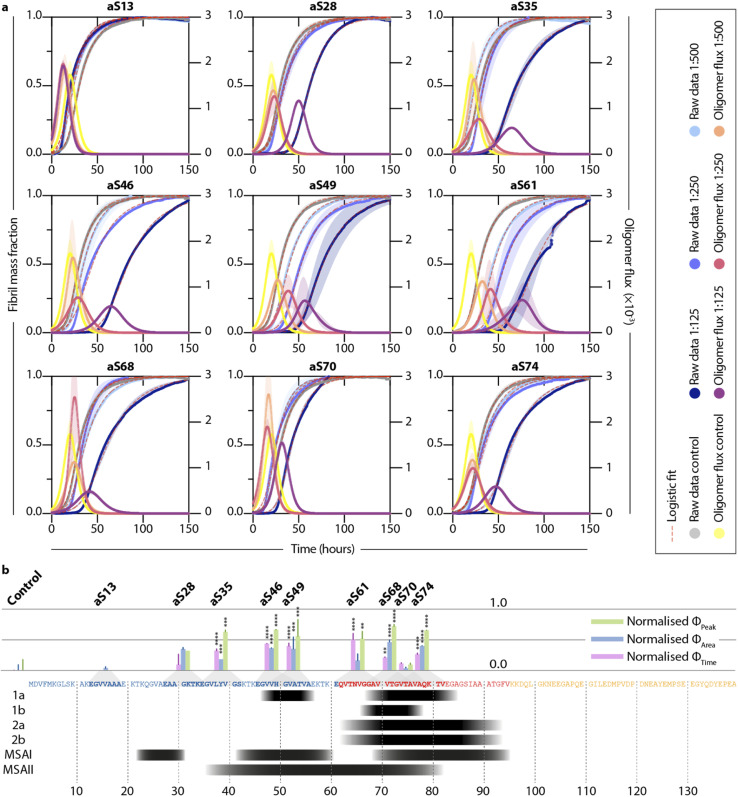
Different DesAbs have distinct effects on the aggregation of α-synuclein. (a) Normalized thioflavin-T (ThT) traces of low seeded aggregations (0.06% preformed seeds in 40 μM monomeric α-synuclein) in the presence of different DesAbs at varying concentrations are shown. Data are fitted with a logistic equation (pink dashed lines). Reactive oligomer flux (*ϕ*) is depicted as characteristic bell shape and is plotted against time (see eqn (7)). Shaded regions correspond to ±s.d. of three technical replicates. (b) Bar graphs showing normalized *ϕ*_Peak_, *ϕ*_Area_ and *ϕ*_Time_ for each DesAb at the highest antibody concentration tested (1 : 125). Lines above the bar correspond to ±s.d. of three technical replicates. The bars are normalized with respect to the control measurement and are displayed at the position of the target epitope along the α-synuclein sequence. The panel below the α-synuclein sequence shows residues which have been discovered to arrange in the core of α-synuclein fibrils using cryo-electron microscopy studies (black). Structures 1–2 refer to different conformations obtained from *in vitro* aggregation reactions, whereas MSAI and MSAII show two folds found in the brains of multiple system atrophy (MSA) patients.^47–50^ Antibody-free aggregation is referred to as control. Data were analyzed using a one-way analysis of variance (ANOVA) and *p*-values are given compared to the mean of the control result. **p < 0.01, ***p < 0.001, ****p < 0.0001.

Next, we tested the DesAbs in a low seeded assay (0.06% of monomeric α-synuclein concentration) (Fig. 3) under mildly acidic conditions to induce secondary nucleation. We found that the DesAbs targeting the region of the α-synuclein sequence that is incorporated closer to the core region of fibrils tended to be more effective at inhibiting the aggregation reaction, in particular aS46, aS49 and aS61. The data also indicate that the scaffold (DesAb-none) and a DesAb designed against Aβ oligomers (DesAb-O) have a minimal effect on the aggregation kinetics (Fig. 2 and S5†). We also checked that the DesAbs do not aggregate themselves (Fig. S6†). Here, X4 and X2 refer to separate batches of DesAb-none. We also conducted transmission electron microscopy (TEM) of the end points from the low seeded aggregation reactions to analyse the morphology of the fibrils. Aggregates from a reaction with and without DesAbs appear similar in length and structure (Fig. S7†), indicating no clear morphological differences, consistent with our finding that the DesAbs have minor effects on elongation. By fitting the aggregation curves obtained from both low seeded and high seeded aggregation assays with the master equation framework, we decoupled the effect of the DesAbs on α-synuclein secondary nucleation from its effect on fibril elongation.

Furthermore, by using the changes in the effective microscopic rate constants, the oligomer flux could be calculated.^33^ This parameter describes the tendency of the system to form oligomeric species over time and is informed by both the apparent rate of secondary nucleation, which acts as a source of oligomeric species, and the apparent rate of elongation, which acts as a “sink” of oligomeric species as they elongate to form mature fibrils. The flux is depicted as a Gaussian function, *ϕ*, for each antibody at a stoichiometry of 1 : 125 and may be used to extract three key parameters relating to the population of oligomeric species, namely, the peak height (*ϕ*_Peak_), area (*ϕ*_Area_) and peak time (*ϕ*_Time_) of the function. These parameters describe the maximum magnitude of the flux with which oligomeric species formation occurs (*ϕ*_Peak_), the total number of oligomers formed over the time course of the aggregation (*ϕ*_Area_), and the time at which the flux towards oligomeric species formation is maximal (*ϕ*_Time_), respectively. Each of the parameters of the function (*ϕ*_Peak_, *ϕ*_Area_, and *ϕ*_Time_) are summarized in a bar plot (Fig. 3b). An ideal DesAb would reduce *ϕ*_Peak_ and *ϕ*_Area_ while maximally delaying *ϕ*_Time_. Our analysis confirmed that aS46, aS49, aS61, aS68 and aS70 have the largest effect on the oligomer flux. These DesAbs are designed to bind to a region that is in the core of α-synuclein fibrils and thus the effect on the oligomer flux to some extent mirrors the structure of α-synuclein fibrils obtained from cryo-EM (Fig. 3b).

Finally, we tested aS46 in an aggregation assay involving the use of lipid vesicles to induce the aggregation of α-synuclein in a neutral pH buffer.^35^ Similar stoichiometries to the seeded assays were used, and the recorded ThT traces indicate that aS46 also affects α-synuclein aggregation under these experimental conditions (Fig. S8†).

Although we were able to identify DesAbs that potently alter secondary nucleation rates in the kinetic aggregation model, it remained challenging to trace these effects back to actual binding events. For instance, DesAbs binding to the catalytic site of α-synuclein fibrils may be the cause of a decrease in secondary nucleation rate. Equally, the DesAbs may also be able to bind to an oligomer's conformation in a secondary nucleation event. While the results obtained from the kinetic analysis provide a good indication of the activity of the antibodies, we sought to further assess the binding to α-synuclein monomers and aggregates in specifically designed binding experiments.

### DesAbs show low binding activity to monomeric and fibrillar α-synuclein

**Fig. 4:**
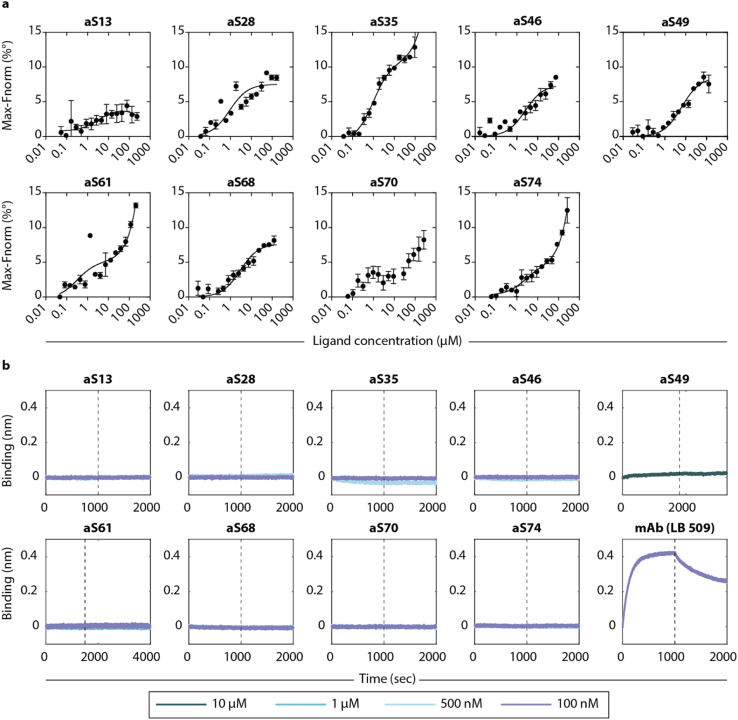
Binding affinities of the DesAbs to monomeric α-synuclein and α-synuclein fibrils. (a) The DesAbs bind monomeric α-synuclein with low μM affinity. Binding curves were obtained from microscale thermophoresis (MST) measurements of fluorophore-labelled α-synuclein and varying concentrations of DesAbs. For better comparison, the position of the lowest signal was set to zero. The binding affinity was also determined at low pH buffer conditions for three DesAbs (see Fig. S13†). Error bars denote ±s.d. of three measurements. (b) Association (before dashed line) and dissociation (after dashed line) to α-synuclein fibrils was recorded with biolayer-interferometry (BLI) for different DesAbs at concentrations as specified. Data were baseline-corrected by subtracting the corresponding baseline from each curve. Well setup and loading traces can be found in Fig. S14.†

To determine the binding affinity to monomeric α-synuclein, we used microscale thermophoresis (MST) with fluorophore-labelled α-synuclein (aSyn-N122C Alexa Fluor™ 488) at varying concentrations of DesAbs. The measurements generated *K*_D_ values in the low μM range (Fig. 4a and Table 1), which are comparable to those found for DesAbs designed to bind Aβ.^27,28^ To test whether a low pH buffer affected the binding affinity, the measurement was repeated at low pH with three of the DesAbs (aS28, aS46, aS68) and resulted in an approximately ten-fold increase in binding affinity towards monomeric α-synuclein. A conformational change of monomeric α-synuclein^36,37^ or a change in charges of the DesAbs leading to a different binding mechanism could potentially explain these results.

**Table 1:** Binding affinities of the DesAbs to monomeric α-synuclein measured by microscale thermophoresis (MST)

Antibody	*K*_D_ (μM)	*R* ^2^
aS13	3.5	0.56
aS28	0.9	0.80
aS35	1.0	0.96
aS46	2.5	0.87
aS49	6.6	0.94
aS61	0.3	0.83
aS68	3.0	0.92
aS70	Fit does not converge	
aS74	2.4	0.96
aS28 (pH 5.5)	0.3	0.96
aS46 (pH 5.5)	0.7	0.98
aS68 (pH 5.5)	0.3	0.96

The binding to α-synuclein fibrils was determined by biolayer interferometry (BLI) by immobilizing α-synuclein fibrils to AR2G biosensors to reduce the effect of avidity in the measurement. No binding signal was observed at DesAb concentrations between 100 nm and 1 μM, as compared to a strong signal obtained from a commercial monoclonal antibody (mAb) (Fig. 4b). The lack of binding signal to fibrils suggest that the epitopes are inaccessible to the DesAbs in a fibrillar conformation (at least in the BLI setup that we used here), which might also be enhanced by the disordered tails of α-synuclein covering the fibrils.^38^ Both experiments are consistent with findings from the kinetic aggregation assay that the DesAbs most likely interact more strongly with conformations of α-synuclein other than the monomeric or fibrillar.

Binding affinity measurements using BLI and MST are powerful high throughput methods for measuring the binding affinities of two isolated binding partners. The measurements work well with a homogeneous molecular target, *e.g.*, monomeric α-synuclein or fibrillar α-synuclein aggregates, since both are bulk measurements. It becomes challenging, however, to quantify affinity when the target species are difficult to isolate or are present in low concentrations. Using classical bulk binding affinity characterization methods prove to be less beneficial for these types of experiments. Firstly, because α-synuclein oligomers have a tendency to be hydrophobic, the aggregates may unspecifically bind to the biosensor or capillary. Furthermore, in order to capture an adequate binding curve for bulk measurements, the experiment needs to use significant amounts and concentrations. A homogeneous cover of the BLI biosensor is desired when filling the device, however this is challenging given the low amounts of α-synuclein oligomers that are formed during an *in vitro* aggregation reaction. Therefore, we next employed single-molecule imaging techniques to study the binding to oligomers.

### Detection of α-synuclein aggregates in PD serum with fluorescence microscopy

We next sought to employ fluorescence microscopy to characterize the binding of the DesAbs to individual α-synuclein aggregates. Seven of the DesAbs (aS13, aS28, aS35, aS46, aS49, aS61, aS68) were labelled with Alexa Fluor™ 647 by *N*-hydroxysuccinimide (NHS) ester chemical conjugation to primary amines after confirming that the complementary epitopes did not contain lysine residues. The serum of PD patients was used to assess the ability of DesAbs to detect α-synuclein aggregates present in these samples.

**Fig. 5:**
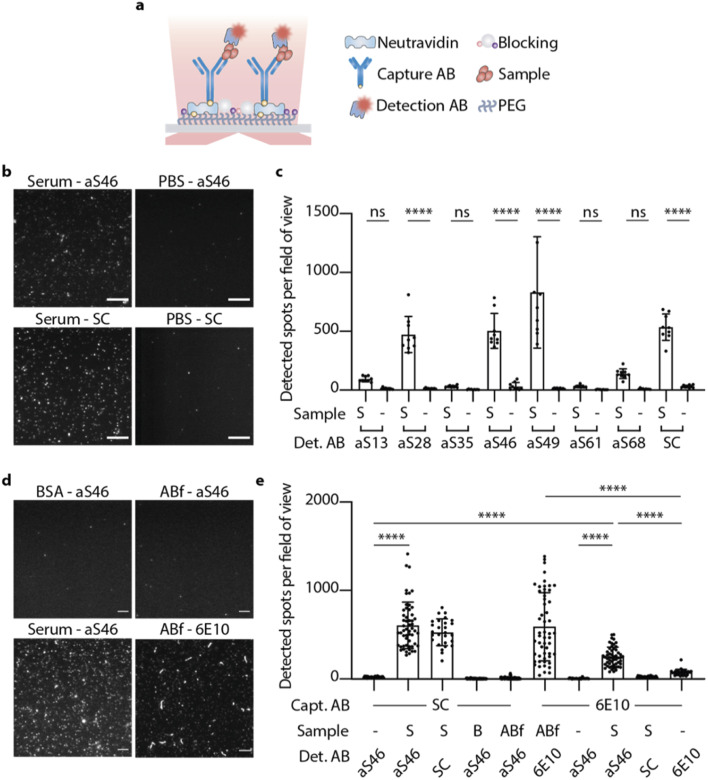
DesAbs bind α-synuclein aggregates in serum samples from PD patients. (a) α-Synuclein aggregates are imaged with the fluorophore-labelled antibody (detection AB) in a single molecule pull down (SiMPull) assay using a capture antibody (capture AB) and TIRF microscopy. (b) Images obtained for fluorophore-labelled aS46 and SC for captured α-synuclein aggregates in serum or a buffer control (PBS); SC was used as capture antibody in all cases; scale bar, 10 μm. (c) Detected spots per field of view for different conditions varying by the sample and detection antibody used; SC was used as capture antibody in all cases. Error bars denote ±s.d. (*n* ≥ 9). Data were analyzed using a one-way analysis of variance (ANOVA). ****p < 0.0001; **p < 0.01; ns, not significant. (d) Images obtained for fluorophore-labelled aS46 and 6E10 for slides with different control samples. In all conditions, SC or 6E10 were used as capture antibody, apart from ABf-6E10, where 6E10 was used as capture antibody. The scale bar indicates 5 μm. (e) Detected spots per field of view for different control conditions (S, serum; B, BSA (50 mg ml^−1^); S, serum; ABf, amyloid β fibrils; -, PBS). Error bars denote ±s.d. (*n* ≥ 27). Data were analyzed using a one-way analysis of variance (ANOVA). ****p < 0.0001.

To specifically detect α-synuclein aggregates in the serum, a single-molecule pull-down (SiMPull) assay was used in combination with total internal reflection fluorescence (TIRF) microscopy.^39,40^ This method has previously been used to study α-synuclein aggregates from human brain tissue using an α-synuclein mAb.^40^ Here, we used a commercial anti-α-synuclein antibody raised against residue 121–125 of monomeric α-synuclein (named SC in the following) acting as capturing antibody immobilized on the coverslip. PD serum samples of three different patients were mixed (1 : 1) for this experiment to balance out a potential variability between patient samples. After incubation with the PD serum and several blocking and washing steps, α-synuclein aggregates were detected to different extents with the fluorophore-labelled DesAbs (Fig. 5). The results are shown in comparison to their corresponding blanks (PBS used instead of PD serum) and the fluorophore labelled SC antibody (Fig. 5b and c). Data for the four different conditions (serum, fibrils, PBS and blank) were obtained by averaging over the intensity of 50 stack images at one field of view (Fig. 5d). Some of the DesAbs showed an increased binding signal compared to their corresponding blank, with aS28, aS46, aS49 and aS68 exhibiting high significance (*p* < 0.0001). The blanks showed essentially no localizations, in contrast to the sample slide detected with either aS46 or SC, which showed remarkably similar distributions of localizations while only the intensity of spots differs. According to how many lysines are available for labeling and therefore the difference in labeling degree between those two types of antibodies, explains the difference in intensity.

All DesAbs showing an increased binding signal were then imaged in an experimental setup where α-synuclein fibrils were captured with SC. Confirming the results obtained using BLI, the DesAbs did not show significantly more binding to α-synuclein fibrils (Fig. S9†) or monomers (Fig. S10†) as compared to the PBS control. SC, however, showed significantly higher amounts of localizations in the sample slide (Fig. S9†) since the SC also binds fibrils. These findings confirm that the DesAbs do not bind fibrils at the concentrations tested and further, that the α-synuclein aggregates present in PD serum are not similar in conformation to *in vitro* α-synuclein fibrils.

To investigate the composition of serum samples and test the specificity of aS46 for α-synuclein aggregates, further experiments with control samples were conducted. We used two types of control samples, a serum mimic composed of bovine serum albumin (BSA) at a concentration similar to that of human serum albumin in human serum (50 mg ml^−1^),^41^ and *in vitro* Aβ fibrils. In both instances, the number of spots per field of view that could be detected was far less compared to the number found in the serum sample (Fig. 5d and e). We also tested whether aS46 shows an increased signal if an antibody specific to Aβ (6E10) was used as capture antibody. Here, fewer spots were observed, but they were nonetheless more numerous than the number of spots found in the PBS sample, while SC detected a level that was comparable to PBS. This finding could be explained by the occurrence of co-aggregates formed from Aβ and α-synuclein monomers,^42^ which would have a different epitope presented than the one SC is targeting. Another possibility is that aS46 binds to a higher extent of non-specifically to certain other species found in human serum. By using a biotinylated version of aS46 as capture antibody, we also tested the extent to which aS46 is able to pull down aggregates from serum and using fluorescently labelled aS46 as detection antibody, we observed an increased number of detected aggregates (Fig. S11b†). We also estimated the binding affinity towards aggregates in serum (*K*_D_ = 41 nM; *R*^2^ = 0.96) by changing the concentration of the detection antibody and fitting a binding curve to the number of detected aggregates for each concentration used (Fig. S11a†).

Finally, we sought to test whether our findings could be applied to other biofluids. We thus analyzed CSF samples pooled from three PD patients, where we detected significantly more α-synuclein aggregates in a SiMPull set-up when using SC as a capture and aS46 as a detection antibody as compared to the PBS blank (Fig. S12†). Notably, the level of α-synuclein aggregates found in PD CSF is about 14-fold lower on average than in PD serum (number of detected spots per field of view: *N*_PD_CSF_ = 27 ± 16 *vs. N*_PD_Serum_ = 370 ± 130), which is consistent with previously reported results.^43^

### Detection of α-synuclein aggregates in PD serum with dSTORM microscopy

**Fig. 6:**
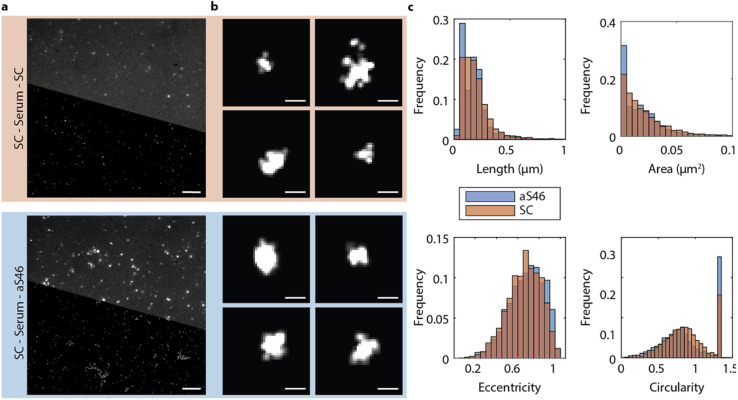
Super-resolution imaging by dSTORM of α-synuclein aggregates of typical size of about 100 nm in serum samples of PD patients. (a) Representative diffraction-limited (top half) and reconstructed super-resolved image (bottom half) of the serum of PD patients in the SiMPull set up. α-Synuclein aggregates were captured with SC and detected with fluorescently labelled SC or aS46. Scale bar, 5 μm. (b) Zoom on super-resolved representative α-synuclein aggregates. Scale bar, 0.1 μm. (c) Length (aS46 mean length = 100 ± 80 nm; SC: mean length = 120 ± 90 nm), area, eccentricity, and circularity distribution of α-synuclein aggregates identified in four separate super-resolved field of views.

The fluorescence spectroscopy method described above could not go beyond the diffraction limit, so we could not use it to establish the size of the α-synuclein aggregates in the PD serum. Therefore, we next analyzed with the DesAbs the size and morphology of the α-synuclein aggregates by direct stochastic optical reconstruction microscopy (dSTORM) imaging, a super-resolution imaging technique. The imaging buffer was changed to a STORM buffer (GLOX buffer, PBS–Tris (50 mM), glucose (0.5 mM), glucose oxidase (1.3 μM), catalase (2.2 μM), and mercaptoethylamine (MEA) (50 mM)), and samples were imaged at a higher laser power and additionally with an activation laser at 405 nm. Thus, the characteristic blinking of fluorophores was achieved, and field of views were imaged for up to 6 min at 33 frames per second. Images were reconstructed using a custom-written Python code accessing the Thunderstorm module in ImageJ. Fig. 6 depicts representative diffraction-limited and reconstructed super-resolved images for serum samples imaged with SC and aS46. We used aS46 since throughout previous characterization it consistently showed strong binding characteristics. Analyzing the length of the α-synuclein aggregates for both conditions revealed a comparable size distribution for both conditions with most α-synuclein aggregates appearing with a length smaller than 200 nm (aS46 mean length = 100 ± 80 nm; SC: mean length = 120 ± 90 nm, *p* < 0.0001 (unpaired *t*-test)) (Fig. 6).

## Conclusions

In this work, we have reported designed antibodies (DesAbs) for the characterization of the conformational properties of soluble aggregates of α-synuclein. The approach is based on the computational design of a panel of antibodies that target epitopes that scan the sequence of α-synuclein, and on their characterization in terms of their ability to bind α-synuclein oligomers. Our results indicate that this approach can lead to the generation of antibodies capable of characterizing heterogeneous populations of soluble α-synuclein aggregates in human serum. We were further able to use one of the designed antibodies in a super-resolution imaging setting and detected α-synuclein aggregates both in serum and CSF from PD patients. Our approach thus offers a route for the development of diagnostic tools for PD and related synucleinopathies.

## Materials and methods

### *In silico* antibody design

The computational method used to identify complementary peptides was described previously.^25,44^ Briefly, complementary peptides are obtained from a library of β-sheet fragments from the protein data bank (PDB). To design a complementary peptide fragments are merged from the PDB library that are found to face segments of the target epitope in a β-strand conformation. This method usually generates more than one complementary peptide for a given epitope. To select the complementary peptide with the best predicted affinity and specificity, DesAbs are ranked based on the following properties of the different fragments: (1) the number of times a pair of fragments is found in native proteins, (2) the length of the fragment, and (3) how many times a fragment faces other peptides which are not in the epitope.

### Protein expression and purification

Plasmids with the corresponding epitope grafted onto the DesAbs scaffold's CDR3 loop were bought commercially (Genscript). In *E. coli* SHuffle T7 competent cells from New England BioLabs, the various antibody plasmids were produced and purified with the addition of ampicillin (100 g ml^−1^). Using Overnight Express Instant TB Medium (Merck Millipore) treated with ampicillin (100 g ml^−1^), cells were cultured overnight for 15 hours at 29 °C and 200 rpm. Cells were collected by centrifugation after an additional hour of incubation at 37 °C, and after being resuspended in 40 ml of TRIS solution that had 10 mM imidazole added, they were sonicated to lyse. Using centrifugation at 18 000 rpm, the supernatant containing the protein was isolated from cell debris (JA-20 rotor, Beckman Coulter). A Ni-NTA Superflow column (Qiagen) that had previously been equilibrated with PBS containing 10 mM imidazole was loaded with the cleared lysate. DesAb that had been his-tagged was eluted in PBS that contained 200 mM imidazole. Finally, employing size exclusion chromatography on a HiLoad 16/600 Superdex 75 column, the protein-containing fractions were combined and further purified (GE Healthcare Life Sciences). By measuring the absorbance at 280 nm and utilizing theoretical extinction coefficients generated by ExPASy ProtParam, the final concentration was computed. Aliquots were kept at −80 °C after being frozen in liquid nitrogen.

### Circular dichroism

A Jasco J-810 spectropolarimeter was used to measure the far-ultraviolet UV CD spectra of several DesAbs using a cuvette with a 0.1 cm-pathlength. At a protein concentration of 10 M, spectra were taken between 200 and 250 nm, at 20 °C, and a baseline of the buffer was systematically subtracted from each spectrum. At a fixed CD wavelength of 207 nm, the temperature denaturation of each DesAb was monitored from 20 to 95 °C at a rate of 0.5 °C min^−1^. The temperature probe was placed inside the cuvette to measure the temperature. The fraction of unfolded protein was used to standardize the measured ellipticity values.

### Preparation of α-synuclein seeds

Monomeric α-synuclein was buffer-exchanged to MES buffer (10 mM 2-(*N*-morpholino)ethanesulfonic acid, 1 mM EDTA, pH 5.5) and concentrated to 200–300 μM using 10 000 kDa MWCO centrifugal spin filters (Amicon Ultra, Millipore). Concentrated α-synuclein monomers were incubated in protein low-binding tubes (Eppendorf) for 72 hours at 40 °C and 400 rpm with a magnetic stirrer. In a benchtop centrifuge, the solution was centrifuged at 21 130 × *g* to determine the fibril concentration (monomeric equivalent) (Eppendorf). By measuring absorbance with a NanoDrop 2000 (Thermo Scientific), the concentration of the remaining -synuclein monomer detected in the supernatant was calculated and subtracted from the initial concentration. After MES buffer was added to the supernatant's volume, the stock was aliquoted and kept at −80 °C. The fibril stock was diluted to a final concentration of 5 μM in protein low binding tubes and sonicated for 15 seconds to create -synuclein seeds.

### Lipid vesicle preparation

2,3-Dimercapto-1-propanesulfonic acid (DMPS) powder was dissolved to a final concentration of 5 mM in 20 mM sodium phosphate buffer at pH 6.5 and stirred at 900 rpm at 50 °C for 2–2.5 h followed by five freeze–thaw cycles in dry ice and a hot water bath at 45 °C to ensure unilamerallity. To form vesicles, the solution was sonicated by a Sonopuls HD 2070 probe sonicator (Bandelin) for 3 × 5 min cycles on a 50% cycle at 10% maximum power, and centrifuged at 15 000 rpm for 30 min at 25 °C to remove residue formed during sonication.

### Plate reader protocol

A Superdex 75 10/300 GL column (GE Healthcare) was used to gel filter purified α-synuclein while it was equilibrated in MES buffer (10 mM 2-(*N*-morpholino)ethanesulfonic acid, 1 mM EDTA, pH 5.5). The peak corresponding to monomeric α-synuclein peptide was then collected in a low-binding test tube (Corning) on ice. Monomeric α-synuclein peptides were used to prepare solutions at a protein concentration of 40 μM in the presence of increasing amounts of the specified DesAb variant in MES buffer supplemented with 5 μM ThT and 0.06% α-synuclein seeds. Then, 150 μl of sample was pipetted into each well of a 96-well half-area plate of black polystyrene with a clear bottom and polyethylene glycol coating (Corning). Evaporation was prevented by sealing the plates. Utilizing a CLARIOstar plate reader (BMG Labtech), aggregation experiments were carried out at 37 °C in quiescent conditions. With a 440 nm excitation filter, the ThT fluorescence was monitored through the plate's bottom at 480 nm every minute.

In the case of vesicle induced aggregation, the measurement was taken with monomeric α-synuclein at a concentration of 20 μM in the presence of increasing amounts of aS46 in MES buffer (10 mM 2-(*N*-morpholino)ethanesulfonic acid, 1 mM EDTA, pH 6.5) supplemented with 5 μM ThT and DMPS at 100 μM at a temperature of 30 °C as previously described.^35^

### Determination of rate constants

In experimental conditions under which α-synuclein fibril elongation is favoured, and where primary and secondary nucleation events are negligible, the initial rate of fibril elongation mat be approximated with a linear function:1
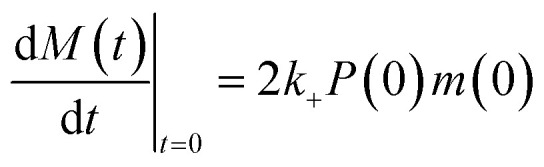
where *M*(*t*) is the fibril mass concentration, *P*(0) is the initial number concentration of fibrils, *m*(0) is the initial monomer concentration and *k*_+_ is the rate of fibril elongation. Since *P*(0) and *m*(0) are universal for each condition tested, the relative rate of elongation for each DesAb may be found by normalising the value of 2*k*_+_*P*(0)*m*(0), that is, the slope of the initial linear points of the elongation reaction, of a given DesAb to that of the control condition.

Under conditions where α-synuclein secondary nucleation is favoured, the aggregation may be described by fitting a generalised logistic function to the normalised aggregation data:2
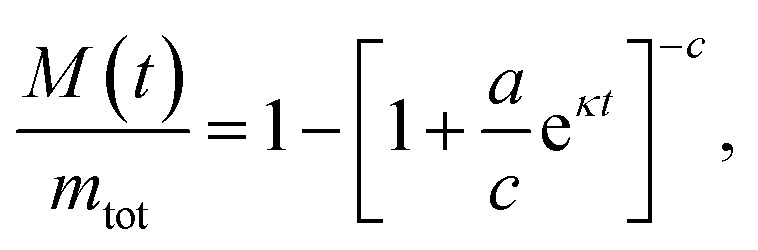
where *m*_tot_ refers to the total α-synuclein monomer concentration present within a reaction. The terms *a*, *κ* and *c* are fitting parameters with3
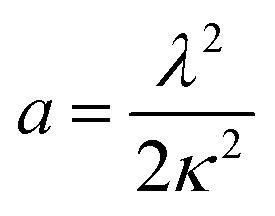
and4
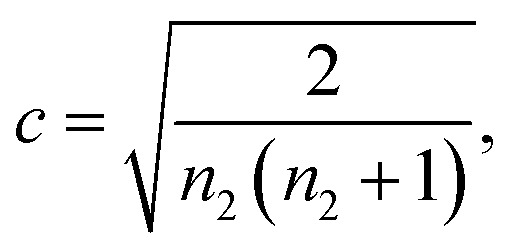
with *c* fixed at a value of 0.3. This corresponds to a reaction order of *n*_2_ = 4, and represents secondary nucleation behaviour as observed in the case of the islet amyloid polypeptide precursor (IAPP) protein.^45^ The terms *λ* and *κ* represent combinations of primary and secondary nucleation rate constants, respectively.^45^

Fitting the generalised logistic function (eqn (2)) to normalised secondary nucleation data provides an analytical approximation for the monomer concentration at time *t*, *m*(*t*), since

5 *m*(*t*) = 1 − *M*(*t*)

This, in turn, facilitates the approximation of the fibril number concentration, *P*, over time by rearranging eqn (1) in terms of the first derivative of *P*(0), which corresponds to the change in fibril number over time, 
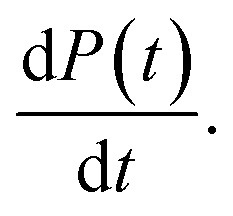
 This is solved at the half-time of each reaction, where it is assumed to me maximal, to yield6
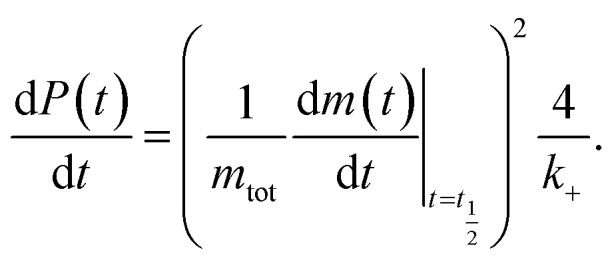

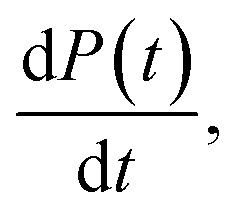
 obtained at the half-time of the reaction, is used as an effective rate of fibril amplification and is reported for each DesAb relative to the control condition.

### Determination of the oligomer flux

Data were fitted as previously described by Staats *et al.*^33^ The flux towards oligomeric species over time, *ϕ*(*t*), was obtained analytically by means of the following equation derived from the linear polymerisation model (eqn (1)):7
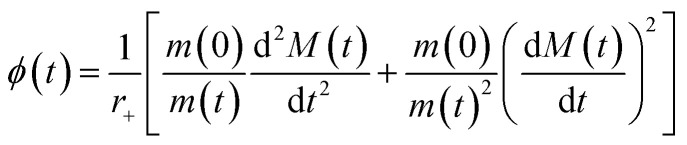
where *M*(*t*) and *m*(*t*) are represented analytically in terms of the fit of the generalised logistic function (eqn (2)) to secondary nucleation data, while *r*_+_ denotes the effective elongation rate obtained for each DesAb as approximated by eqn (1).

To approximate the parameters of the flux towards oligomeric species, eqn (2) may be substituted into eqn (7) to yield an expression for *ϕ*(*t*) in terms of the generalised logistic function:8
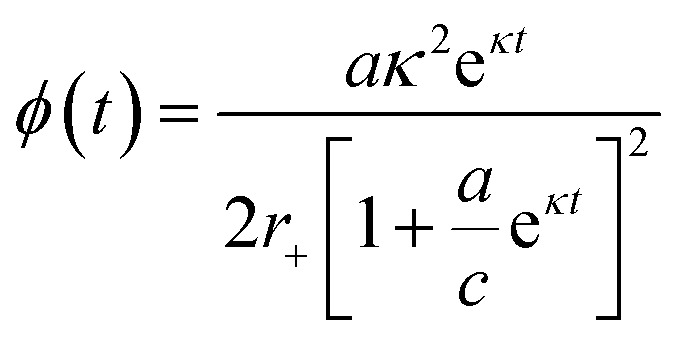
from which the area under the curve of the flux towards oligomeric species, *ϕ*_Area_, may be calculated by calculating the integral of eqn (8):9
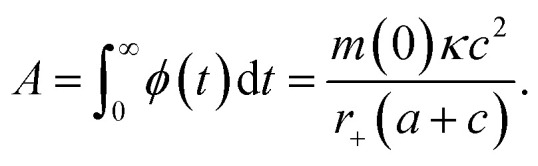


Eqn (8) may also be used to approximate the peak time and peak height of *ϕ*(*t*), since the peak time may be calculated as a solution to10
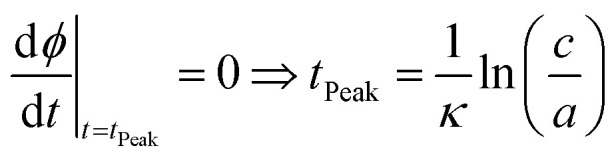
which yields a description for the peak height of *ϕ* as11
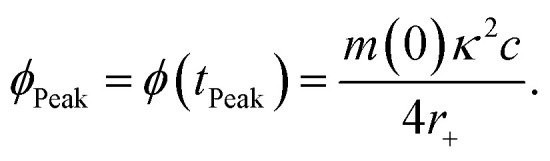


The extracted values for *ϕ*_Peak_, *ϕ*_Area_, and *ϕ*_Time_ of each condition, *i*, were then normalised to the corresponding mean value for three replicates of α-synuclein only (control):12
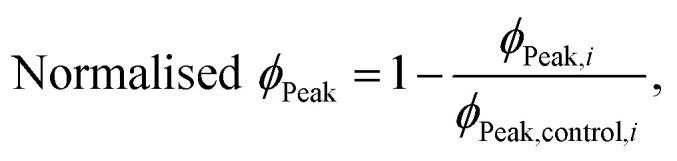
13
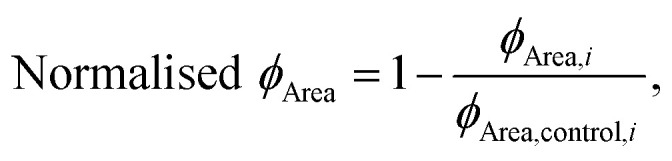
14
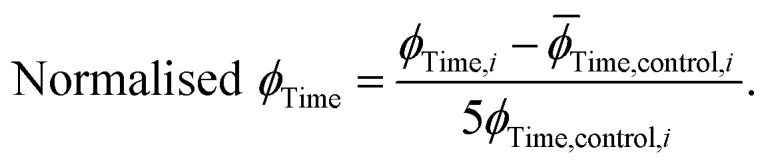


### Microscale thermophoresis (MST)

MST was measured using a Nanotemper Monolith NT.115 instrument using Monolith NT Standard Treated Capillaries (NanoTemper Technologies). Using 10 K Ultracel Amicon Ultra-15 centrifugal filters, purified DesAbs were concentrated and buffer exchanged to a final concentration of 90–150 μM in MST buffer (50 nM Tris–HCl, 150 nM NaCl, pH 7.4). DesAbs were serially diluted 1 : 1.75 to produce 16 solutions of 15 μl in MST buffer. After making a stock solution of MST buffer containing 10% Tween 20, α-synuclein was diluted into the solution to a final concentration of 200 nM and 0.2% Tween 20. Then, 50 nM of the diluted α-synuclein and 0.05% Tween 20 were added to each serial dilution solution. After performing a capillary scan and loading 10–15 μl of solution into each capillary, the thermophoresis was measured. The optimal parameters were 50% LED power (blue), 80% MST power at a temperature of 22 °C, 5 s fluorescence before MST activation, 20 s MST activation, 5 s fluorescence after MST activation, and a 25 s delay between runs. Before each MST scan, a capillary scan and a capillary position scan were performed to check for unspecific protein binding to the capillary and proper sample preparation, respectively. Using Prism (Graphpad) and the saturation binding equation *Y* = (*B*_Max_ × *x*)/(*K*_D_ + *x*), where *B*_Max_ is the maximal amount of specific binding, equilibrium dissociation constants, *K*_D_, were calculated.

### BLI measurements

Amine reactive second generation (AR2G) biosensors from FortéBio were used for the measurements on the BLI platform Octet K2. 200 μl were used for each well's sample volume. Biosensors were soaked in PBS for at least 10 minutes in a 96-well plate prior to each cycle. Initially, the pH of the buffer and the concentration of -synuclein fibrils were changed to provide the best immobilization conditions for -synuclein aggregates. 5 μM α-synuclein fibril concentration was determined to be the ideal condition (pH 7.4). PBS was utilized as an equilibration buffer and baseline buffer. Biosensors were activated using a freshly prepared EDC/sNHS mix (20 mM EDC and 10 mM sulfo-NHS in 10 mM acetate buffer, pH 5) before the plate was set up. Tips were quenched in 1 M etanolamine after being loaded with -synuclein fibrils (pH 8.5). A baseline was recorded before each sample's association was evaluated. One tip served as a reference by having PBS in the matching well instead of immobilized -synuclein to control for non-specific antibody binding to the biosensor. Reference curves were subtracted from sample curves and plotted using a custom-written code in order to examine binding of each DesAb (MATLAB).

**Table 2:** Demographic information of Parkinson's disease cases used in this work. ACE-III, Addenbrooke's Cognitive Examination-III; LBD, Lewy body dementia; MDS-UPDRS, movement disorder society unified Parkinson's disease rating scale; PD, Parkinson's disease; PMI, post-mortem interval

Sample number	Patient ID	Gender	Age (years)	PD duration (years)	MDS-UPDRS total	MDS-UPDRS motor score	H & Y	ACE-III
PD13	PDP1335	Female	64.05	2.06	N/A	N/A	2	100
PD14	PDP1373	Male	60.54	2.51	28	16	2	92
PD15	PDP1308	Male	68.07	3.44	N/A	N/A	2	98
PDC2	PDP1395	Male	69.61	0.82	51	28	2	95
PDC3	PRP1388	Female	78.04	0.59	58	40	2	97.3
PDC5	PDP1390	Female	78.87	0.34	37	30	2	89

### Serum and CSF samples

Serum and CSF samples were provided from patients with idiopathic PD, meeting UK PD Brain Bank diagnostic criteria, who were participants in a University of Cambridge study investigating the role on inflammation and protein aggregation in the development of PD dementia (NET-PDD: Neuroinflammation and Tau Aggregation in Parkinson's Disease Dementia). All participants provided informed consent and ethical approval was given by the East of England-Essex Research Ethics Committee (16/EE/0445). Demographic and clinical data were available (Table 2), matched to the time of sample collection, including disease severity (Movement Disorder Society Unified Parkinson's Disease Rating Scale (MDS-UPDRS)), disease stage (Hoehn and Yahr scale) and cognitive status (Addenbrooke's Cognitive Examination Version 3 (ACE-III)). Participants were within 4 years of diagnosis and were non-demented.

Venous blood samples were collected in 7.5 ml S-Monovette tubes (Table 1) before leaving them to clot at room temperature for 15 min followed by 15 min of centrifugation at 2000 rpm. The supernatant (serum) was aliquoted and stored at −80 °C until use.

Lumbar puncture was performed under sterile technique with 1% lignocaine local anesthetic (Table 1). 5 ml of CSF was collected and centrifuged for 10 min at 300 G at 4 °C before samples were aliquoted and stored at −80 °C.

Before imaging a 1 : 1 dilution of serum or CSF samples as listed in Table 1 was prepared.

### Preparation of *in vitro* α-synuclein fibrils for SiMPull

Monomeric recombinant α-synuclein was incubated at a concentration of 200 μM in PBS for 72 hours at 37 °C with constant agitation of 200 rpm in an orbital shaker (Eppendorf). Before imaging fibrils were diluted to a final concentration of 500 nM monomer equivalent.

### Preparation of *in vitro* Aβ for SiMPull

Monomeric recombinant Aβ (Abeta42, rPeptide) was incubated at a concentration of 4 μM in PBS for 8 hours at 37 °C. Before imaging fibrils were diluted to a final concentration of 500 nM monomer equivalent.

### Fluorescence microscopy

#### Sample preparation for fluorescence microscopy

All TIRF experiments were conducted using covalently PEGylated glass coverslips. Coverslips were prepared following a previously published protocol,^46^ with minor modifications. The glass cover slips (26 × 76 mm, thickness 0.15 mm, Thermo) were cleaned using an ultrasonic cleaner (USC100T, VWR) in a series of solvents for 10 minutes each: 18.2 MΩ cm MQ water, acetone, and MeOH. After being washed, the cover slips were etched with 1 M KOH under 20 minutes of ultrasonication, rinsed with MeOH, 18.2 M MΩ cm MQ water, and dried with nitrogen flow before being cleaned with argon plasma for 15 minutes (Femto Plasma Cleaner; Diener Electronic). Clean coverslips were salinized for 20 min with 60 s of ultrasonication at the beginning and middle of the process using 5 ml of 3-aminopropyl triethoxysilane (Fisher Scientific UK, cat. no. 10677502), 8.3 ml AcOH in 166.7 ml MeOH (10 min after the start point). Following a rinse in MeOH, 18.2 MΩ cm MQ water, and MeOH, the salinized coverslips were dried with nitrogen flow. The coverslips were then cleaned and salinized, and 50-well PDMS gaskets (Sigma, GBL103250-10EA) were afterwards applied. A 100 : 1 aqueous combination of MPEG–SVA-5000 (110 mg ml; MW 5000; Laysan Bio Inc.) and biotin–PEG–SVA-5000 (1.1 mg ml; MW 5000; Laysan Bio Inc.) was added to the wells to passivate them, along with 1 ml of 1 M NaHCO_3_ (pH 8.5). In a humid chamber, the coverslips were incubated with PEG solution for an entire night. After being rinsed with 18.2 MΩ cm MQ water, they were dried with nitrogen flow. In order to further treat the passivated wells, 9 μl of MS(PEG)4 methyl-PEG–NHS–ester (10 mg ml^−1^, Thermo Fisher, cat. no. 22341) and an additional 1 μl of 1 M NaHCO_3_ (pH 8.5) were added. The coverslips were placed in a humid chamber for an overnight PEG solution incubation, rinsed with 18.2 MΩ cm MQ water, and then dried with nitrogen flow. Glass coverslips that had been PEGylated were kept in a desiccator at −20 °C until needed.

Before slide preparation, the following three buffers for washing, dilution and imaging were prepared (all at pH 7.4): 1 × PBS (PBS), 1 × PBS + 0.05% Tween 20 (PBS–T), 1 × PBS + 0.05% BSA + 0.0.5% Tween 20 + 10% salmon sperm (PBS–B). 10 μl of NeutrAvidin (0.2 mg ml^−1^) was added to each well and was left to incubate for 10 min. Each well was then washed with PBS–T three times before adding 10 μl of the biotinylated capture antibody sc-12767 (SC, Santa Cruz) at 12.1 nM for 15 min. With PBS–T, the extra antibody was removed three times. The target sample was then added to the well, such as a 500 nM fibril sample or an undiluted serum sample, and allowed to incubate for 60 min. PBS was placed into blank wells for the same amount of time. Following the incubation, samples were taken out of the wells and each well received 10 μl PBS–B for a 60 minute blocking step, followed by three PBS–B–W washes. The detection antibody was added at a dilution of 2 nM (DesAb) or 5 nM (mAb) and incubated for 30 min (DesAbs) or 10 min (mAb) (mAb). The wells were then filled with 3 μl of PBS and sealed with a plasma-cleaned cover slip after being washed with PBST five times in total.

#### TIRF imaging

Imaging was carried out using a home-built total internal reflection fluorescence (TIRF) microscope consisting of a Ti-E Eclipse inverted microscope (Nikon) with an oil-immersion objective (UPlanSApo, 100×, 1.49 NA; Olympus) and a perfect focus system. AF647 was excited with a 638 nm laser (iBeam-Smart, Toptica) and coupled into the sample by a dichroic mirror (Di01-R405/488/561/635, Semrock). Emission was collected by the objective lens and separated from excitation light using a dichroic mirror (Di01-R405/488/561/635; Semrock) and passed through appropriate emission filters (FF01-692/40-25; Semrock). The emitted fluorescence was then focused onto an air-cooled EMCCD camera (Photometrics Evolve, EVO-512-M-FW-16-AC-110). Image stacks of 50 frames were acquired with an exposure time of 50 ms. To minimize any bias associated with the ROI selection, an automated script (Micro-Manager) was used to collect images in a grid. Images were analyzed using a custom-written ImageJ script analyzing the maxima in each image.

#### dSTORM imaging

For single molecular super-resolution imaging, the sample slides were prepared as described above and after diffraction-limited imaging was completed the imaging solution was removed. Three additional sheets of 50-well PDMS gaskets (Sigma, GBL103250-10EA) were then connected to the first sheet in order to retain a larger volume in each well. Then, mercaptoethylamine (MEA) (50 mM) was pipetted into each well along with PBS–Tris (50 mM), glucose (0.5 mM), glucose oxidase (1.3 μM), catalase (2.2 μM), and dSTORM buffer (50 mM). Before imaging, MEA was immediately added to the buffer. By stimulating the sample with the 638 nm laser at 150 mW laser power while also running the 405 nm laser at 20 mW power, image stacks of 8000 frames were captured at 33 frames per second with an exposure duration of 30 ms. The resulting image had 105.4 nm for each pixel. Super-resolution images were reconstructed using a custom-written Python script with the Fiji package Thunderstorm.

## Data availability

Additional data supporting this manuscript is included within the associated ESI.†

## Author contributions

K. K., M. V. and D. K. designed the experimental plan. P. S. and F. A. A. provided antibody designs and gave advise during experimental part of the work. K. K. performed protein preparation and biophysical characterization of antibodies. D. E., Y. Z. and E. L. optimized fluorescence imaging conditions. K. K., D. E. and Y. Z. conducted fluorescence microscopy experiments. Y. Z. provided super-resolution image analysis code. K. K and R. S. conducted kinetic aggregation assay experiments. R. S. performed computational analysis of kinetic aggregation data and performed TEM experiments. K. K. and L. S. conducted BLI experiments. A. K. prepared human serum and CSF samples. O. R. gave advice during antibody design and protein preparation. K. K., D. E., R. S., O. R., C. W., D. K. and M. V. wrote the article. D. K., M. V. and C. W. were responsible for funding acquisition.

## Conflicts of interest

There are no conflicts to declare.

## Supplementary Material

SC-013-D2SC00066K-s001
