# Supplementary material for: An antibody scanning method for the detection of α-synuclein oligomers in the serum of Parkinson's disease patients
Source: Chem Sci. 2022 Sep 22;13(46):13815–28. doi: 10.1039/d2sc00066k (PMC9710209; doi:10.1039/d2sc00066k)
Supplement: SC-013-D2SC00066K-s001 [file SC-013-D2SC00066K-s001.pdf]

Electronic Supplementary Material (ESI) for Chemical Science.
This journal is © The Royal Society of Chemistry 2022

aS13
```
index_of_first_seq_res: 12 position 13 len=8
K||E|G|V|V|A|A|A|E||K   <---> RQIVSAR antiparallel
(CamSol(range)=1.687232)
  |:   :   :   :  | :           -=+-+ (ME=1.270378)
 || | |I|V|S|A|R| ||   count= 1  promiscuity=  0
V||R|Q| | | | | | ||   count= 2  promiscuity= 35
 || |Q|I|V| | | | ||   count= 1  promiscuity=165
 || | | | | | |R|I||V  count= 1  promiscuity=195
VV|R| | | | | ||   count= 1  promiscuity=391
```

aS28
```
 index_of_first_seq_res: 27 position 28 len=8
A||E|A|A|G|K|T|K|E||G   <---> REANYNEI antiparallel
(CamSol(range)=2.092692)
  |:   :   :   :  | :           -+=-++- (ME=1.420449)
 || | | |N|Y|N|E|I||   count= 1  promiscuity=  0
M||R|E| | | | | | ||   count= 1  promiscuity=  9
 || |E|A|N| | | | ||   count= 3  promiscuity= 39
```

aS35
```
 Targeting REGION P1 of Sheena Radford paper:
 index_of_first_seq_res: 34 position 35 len=8
K||E|G|V|L|Y|V|G|S||K   <---> RITAVGHV antiparallel
(CamSol(range)=0.869799)
: |  :   :   :   :|             +=-+-+=- (ME=1.114625)
 || | |T|A|V|G|H|V||   count= 1  promiscuity=  0
Y||R|I| | | | | | ||   count= 1  promiscuity= 85
 ||R|I|T| | | | | ||   count= 1  promiscuity=240
```

aS46
```
 index_of_first_seq_res: 45 position 46 len=8
K||E|G|V|V|H|G|V|A||T   <---> RILSGVYL antiparallel
(CamSol(range)=0.657795)
: |  :   :   :   :|             +=+-=+-+ (ME=1.163247)
 || | | |S|G|V|Y|L||V  count= 1  promiscuity=  0
Y||R|I| | | | | | ||   count= 1  promiscuity= 85
 || |I|L|S| | | | ||   count= 1  promiscuity=275
 || |I|F|S| | | | ||   count= 1  promiscuity=123
 ||N|I|L| | | | | ||   count= 1  promiscuity=141
 || |I|M|S| | | | ||   count= 1  promiscuity= 35
 ||K|I|P| | | | | ||   count= 1  promiscuity= 46
 || |I|P|S| | | | ||   count= 1  promiscuity= 27
L||R|A| | | | | | ||   count= 1  promiscuity=207
 || |A|V|S| | | | ||   count= 1  promiscuity=230
 ||K|L|V| | | | | ||   count= 1  promiscuity=298
 || |L|V|S| | | | ||   count= 1  promiscuity=245
```

aS49
```
 index_of_first_seq_res: 48 position 49 len=8
E|V||V|H|G|V|A|T|V|A||E   <---> SGVYLVGE antiparal-
lel (CamSol(range)=0.615874)
    |:   :   :   :  | :           -=+-+-+-
(ME=0.933835)
   ||S|G|V|Y|L|V|G| ||   count= 1  promiscuity=  0
+ -|| | | | | |V|G|E||   count= 3  promiscuity= 79
N|Q||S| | | | | ||   count= 1  promiscuity= 15
```

aS61
```
  index_of_first_seq_res: 61 position 62 len=8
E||Q|V|T|N|V|G|G|A||V   <---> QYSVLIDADV antiparallel
(CamSol(range)=0.809011)
: |  :   :   :   :|             =---+=+- (ME=0.918387)
Q||Y|S|V|L|I| | | ||   count= 1  promiscuity=  0
 || | | | |I|D|A| ||   count= 2  promiscuity= 77
 || | | | | | |A|D||V  count= 1  promiscuity= 78
```

aS68
```
 G||G|A|V|V|T|G|V|T|A   <--->  YLQSVAEQL antiparallel
(CamSol(range)=1.246822)
  |:   :   :   :  |:            ++=--+-+ (ME=1.059429)
 || | | |S|V|A|E|Q|   count= 1  promiscuity=  0
S||Y|L| | | | | | |   count= 1  promiscuity=170
 ||Y|L|Q| | | | | |   count= 1  promiscuity=141
 || |L|Q|S| | | | |   count= 2  promiscuity= 49
         | | | | |Q|L||S| |  count= 1  promiscuity=110
         | | | | | | ||S|I|F count= 1  promiscuity=104
         | | | | | |L||S|I|  count= 1  promiscuity=280
       | | | | | |Q|L||T|   count=12  promiscuity=216
       | | | | | | |L||T|V  count= 2  promiscuity=1084
```

aS70
```
  index_of_first_seq_res: 70 position 71 len=8
V||V|T|G|V|T|A|V|A||Q   <---> YGHGIGHEK antiparallel
(CamSol(range)=1.944163)
: |  :   :   :   :|             =++=-=-- (ME=1.012048)
 || | | |I|G|H|E|K||   count= 2  promiscuity=  0
Y||G|H| | | | | | ||   count= 1  promiscuity= 13
 ||G|H|G| | | | | ||   count= 1  promiscuity= 11
 || | |G|I|G| | | ||   count= 1  promiscuity= 90
```

aS74
```
  index_of_first_seq_res: 73 position 74 len=8
G||V|T|A|V|A|Q|K|T||VE   <---> LEIKAKFTV antiparallel
(CamSol(range)=1.818182)
  |:   :   :   :  | :           ++=-++-++
(ME=1.175002)
 || |E|I|K|A|K| | ||   count= 1  promiscuity=  0
E||L|E| | | | | | ||   count= 1  promiscuity=208
 || | | | | |K|F|T||   count= 1  promiscuity=193
 || | | | | | |F|T||A  count= 1  promiscuity=216
 ||L|E|I| | | | | ||   count= 1  promiscuity=347
 || | | | | |K|F|T||V  count= 1  promiscuity= 18
   | | | | | | |T||VK  count= 4  promiscui-
ty=417(CamSol(range)=0.836080)
```

Figure S1. Antibody designs generated with the cascade method. Details of the sequence fragments used to build the complementary peptides are shown.

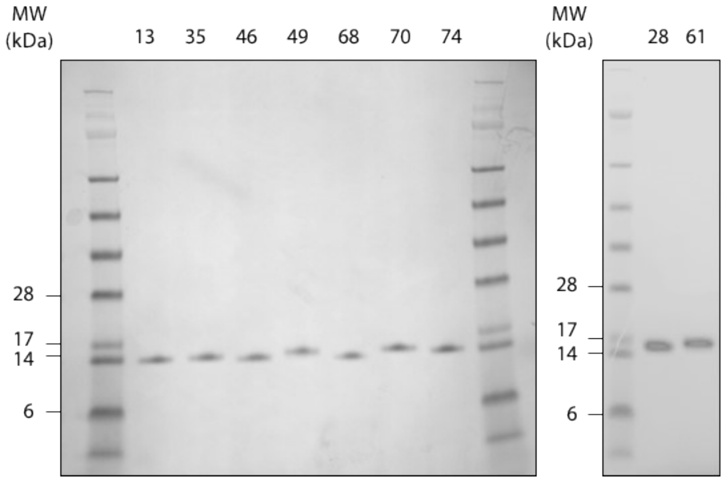

Figure S2: SDS-PAGE analysis of purified DesAbs. Labels at the well entry denote the DesAb name. MW, molecular weight; kDA, kiloDalton.

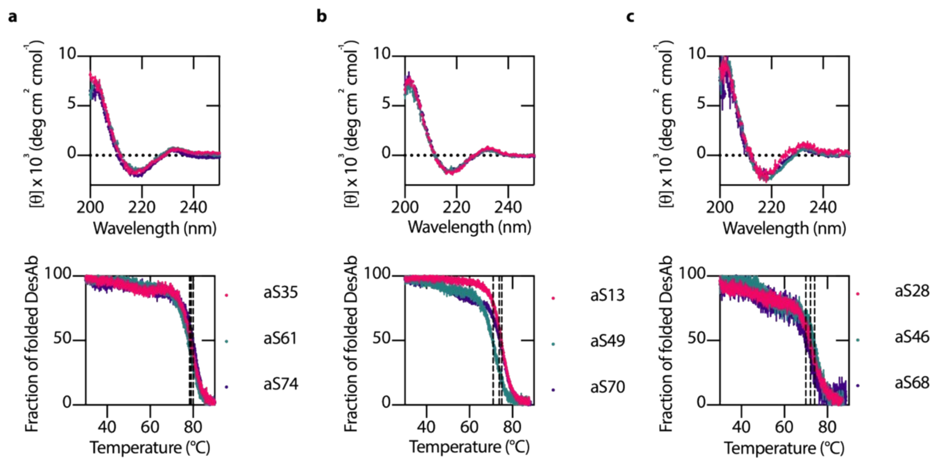

Figure S3: Thermostability and structure of designed antibodies against α-synuclein. Circular dichroism (CD) spectra using a constant temperature of 22 °C (upper panels)**,** and thermal denaturation at temperatures ranging from 20 °C to 90 °C was recorded at a constant wavelength of 207 nm (lower panels). Error bars represent +/- s.d. of three technical repeats. [θ] mean residue ellipticity.

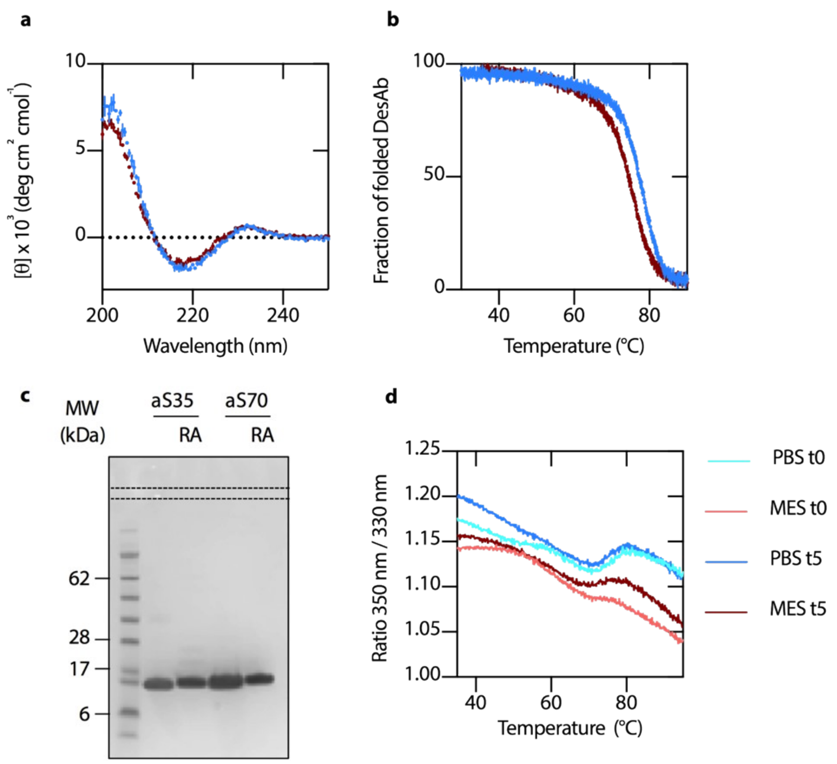

Figure S4: Thermal stability of the DesAbs. aS61 was buffer exchanged to MES buffer (10 mM 2-(N-morpholino)ethanesulfonic acid, 1 mM Ethylenediaminetetraacetic acid (EDTA), pH 5.5) and incubated for 5 days at 37 °C under quiescent conditions as used in the α-synuclein aggregation assay. The antibody conformation was conserved as shown in: CD spectra **(a)**, thermal denaturation curve measured using CD **(b)**, and thermal denaturation measured using a Tycho NT.6 system **(d)**. DesAbs measurements taken at time T0 and time T5 correspond to the beginning and end of the incubation, respectively. Error bars represent ± s.d. of three technical repeats. [θ], mean residue ellipticity. **(c)** SDS-PAGE analysis of two DesAbs which have been incubated in MES buffer at 37 °C for five days. Larger molecular weight species and a band at the entry of the wells (dashed lines) are not present indicating that DesAbs are not aggregating at these conditions. MW, molecular weight; kDa, kiloDalton; RA, reducing agent.

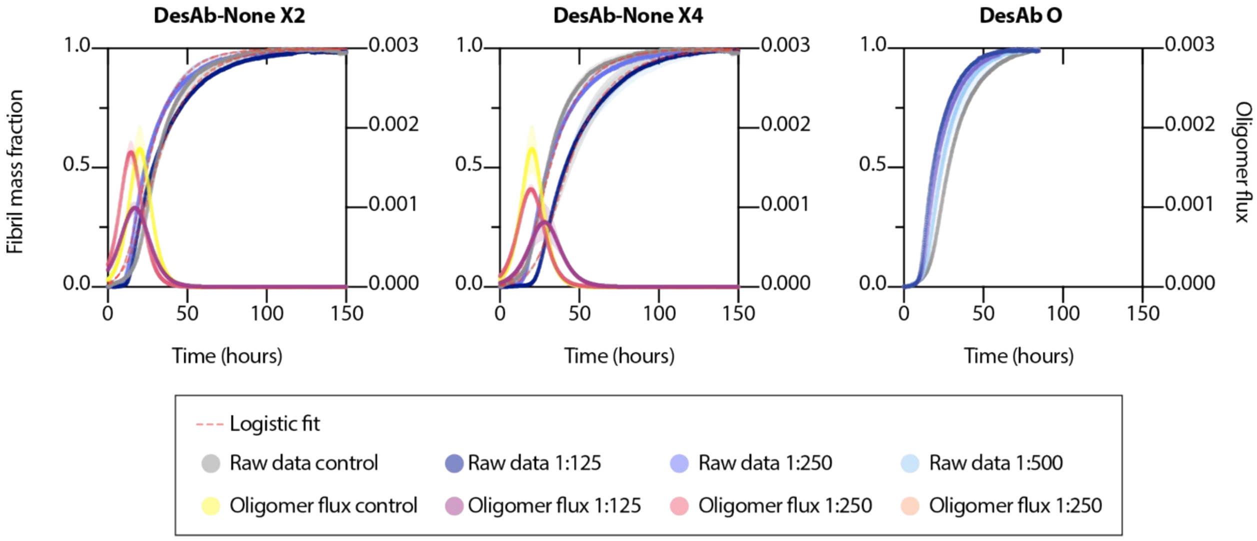

Figure S5: DesAb lacking an engrafted paratope (DesAb-None) and DesAb against Aβ do not inhibit the aggregation of α-synuclein. Normalized ThT traces of seeded aggregation assays (0.06% preformed seeds in 40 μM monomeric α-synuclein in the presence of different DesAbs at variable concentrations. A logistic equation is used to fit the data (pink dashed lines). Reactive oligomer flux (ϕ) is depicted as characteristic bell shape and is plotted against time (see Eq. 7). Shaded regions correspond to ± s.d. of three technical replicates.

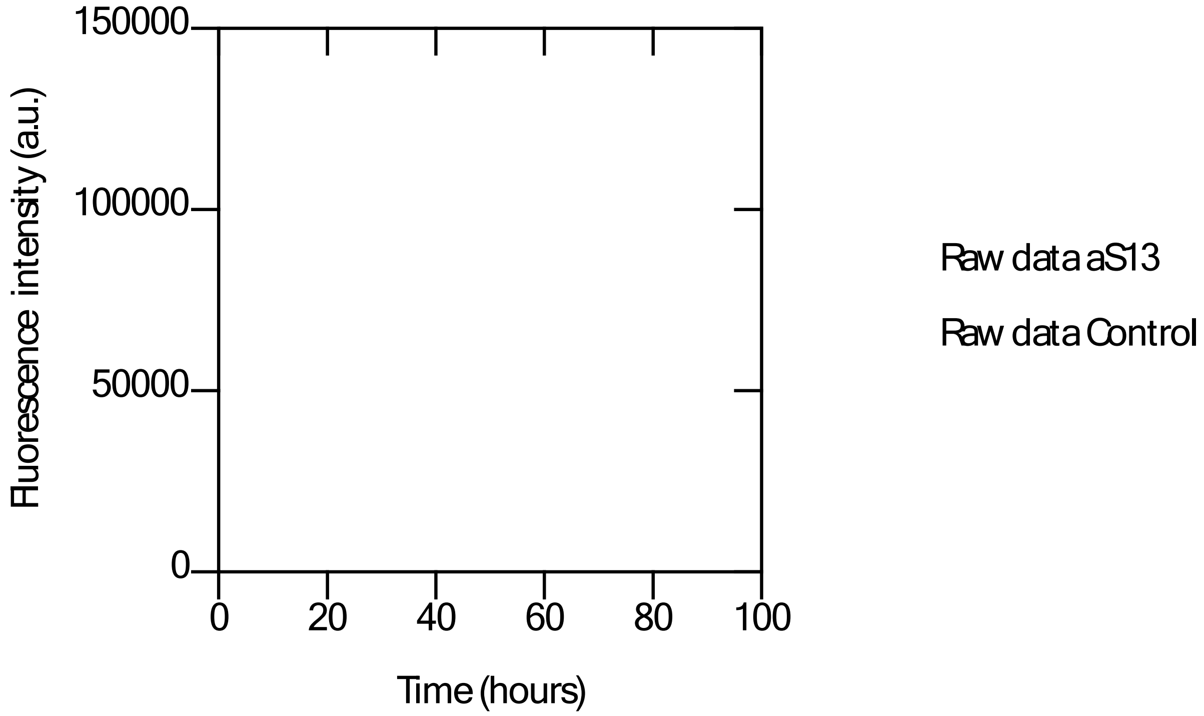

Figure S6: The ThT fluorescence is not affected by the presence of DesAb aS13. ThT traces in the presence of 320 nM DesAb aS13 (blue) or seeded α-synuclein (0.06% preformed seeds in 40 μM monomeric α-synuclein; brown). Shaded areas correspond to ± s.d. of three technical replicates.

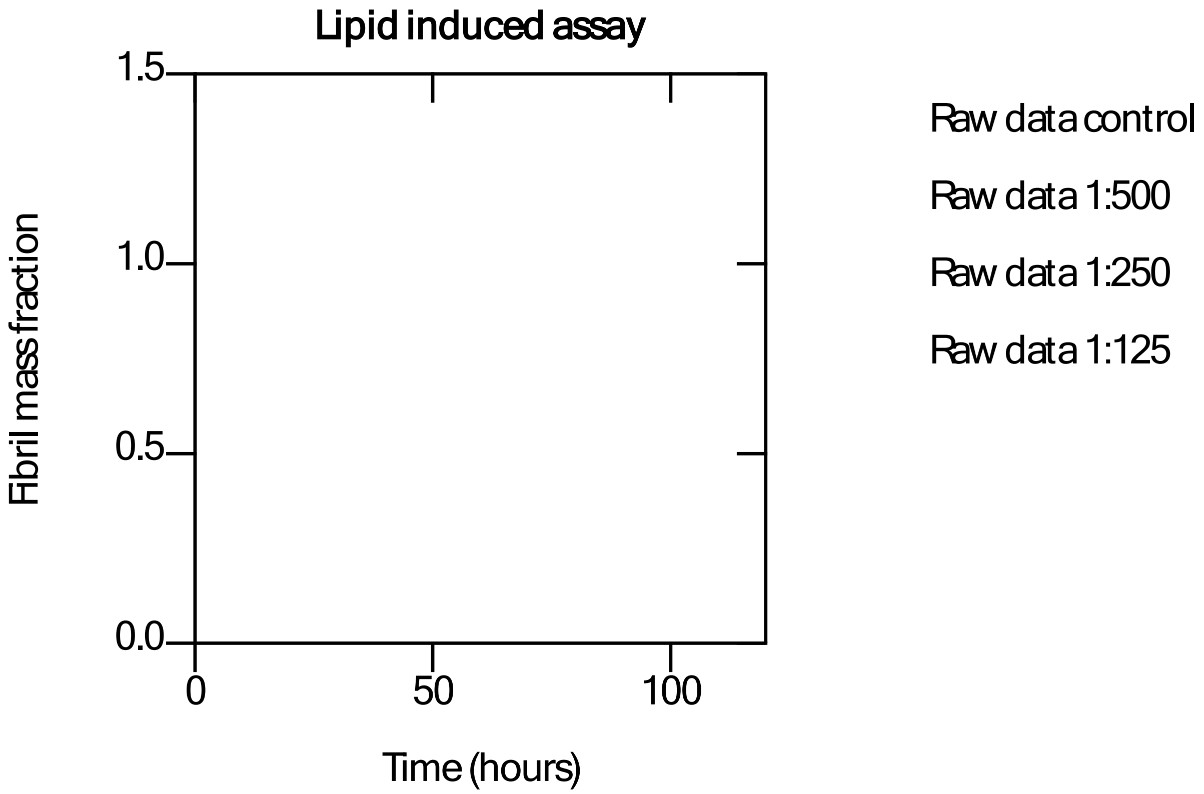

Figure S7: DesAb aS46 affects the aggregation of α-synuclein at neutral pH in the presence of lipid vesicles. Normalized ThT traces in the presence of lipid vesicles (100 μM DMPS in 20 μM monomeric α-synuclein) and of DesAb aS46 at varying concentrations. Shaded areas correspond to ± s.d. of two technical replicates.

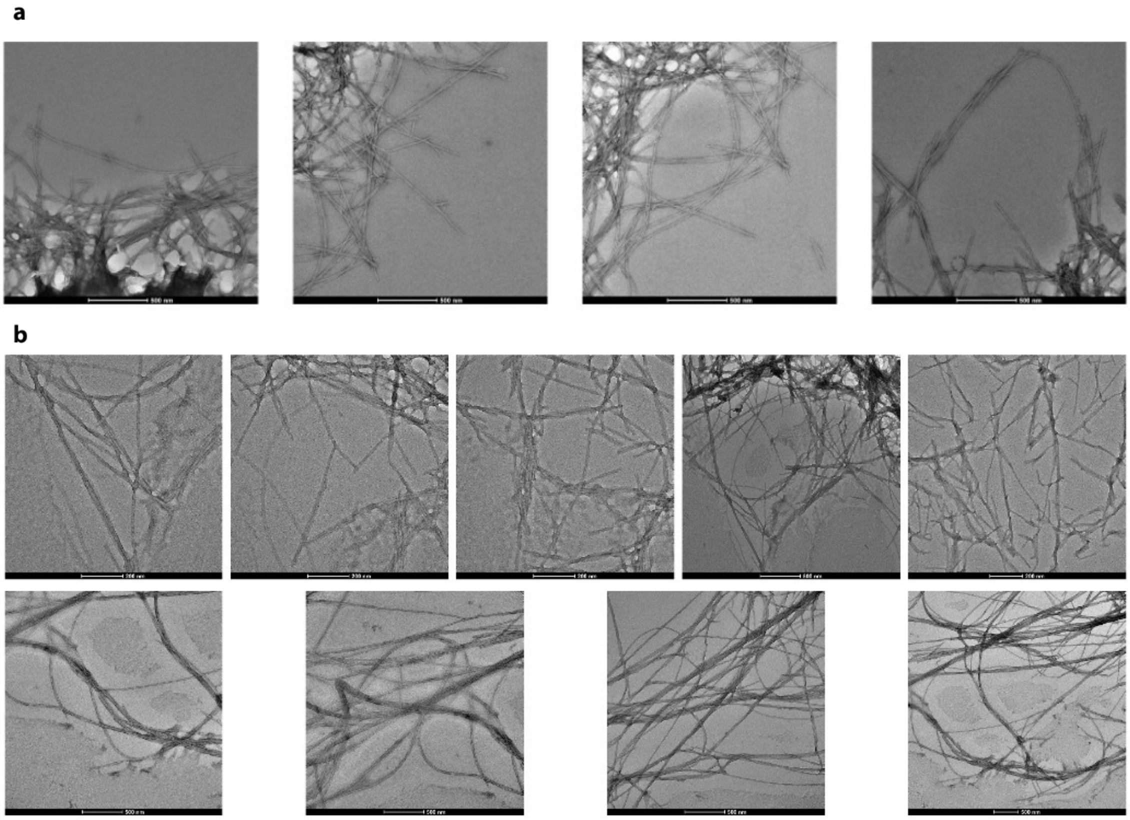

Figure S8: TEM images of α-synuclein fibrils. TEM images from an α-synuclein aggregation (low seed, 0.06% preformed α-synuclein seeds, 40 μM α-synuclein monomer, MES buffer, pH 5.5) (a) without the presence of DesAb (Control), and (b) in the presence of 1:500 DesAb aS46 relative to monomer. Scale bar, 500 nm.

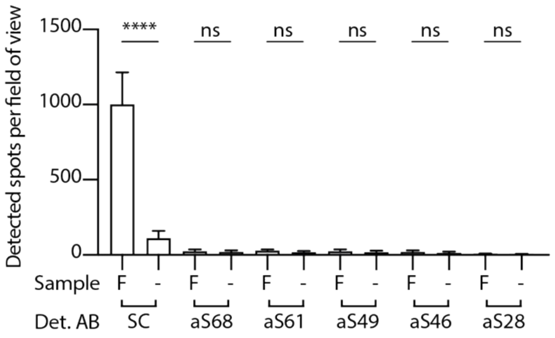

Figure S9: DesAbs do not detect α-synuclein fibrils in a SiMPull assay. Detected spots per field of view for different conditions varying by the detection antibody used. Error bars denote ± s.d. ($n > 9$). Data were analyzed using a one-way analysis of variance (ANOVA). **** $p < 0.0001$; ns, not significant. Det. AB, detection antibody; SC, Santa Cruz antibody; F, fibrils; -, PBS control.

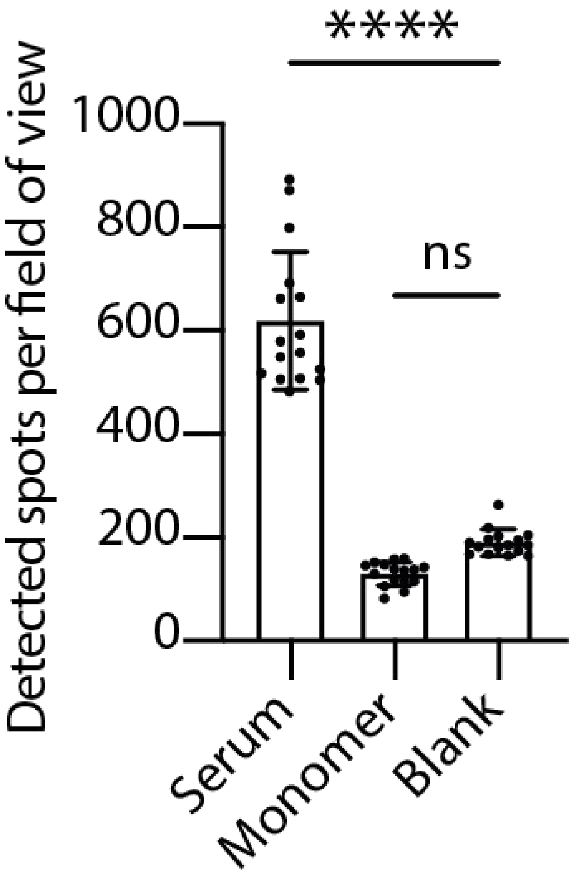

Figure S10: DesAb aS46 selectively detects α-synuclein oligomers in PD serum. The number of detected spots per field of view is shown for a SiMPull experiment in which the SC antibody was used as a capture antibody, PD serum, recombinant monomeric α-synuclein (500 nM), or PBS used as a sample and DesAb aS46 as a detection antibody. Error bars denote ± s.d. ($n > 4$). Data were analyzed using a one-way analysis of variance (ANOVA). **** $p < 0.0001$; ns, not significant.

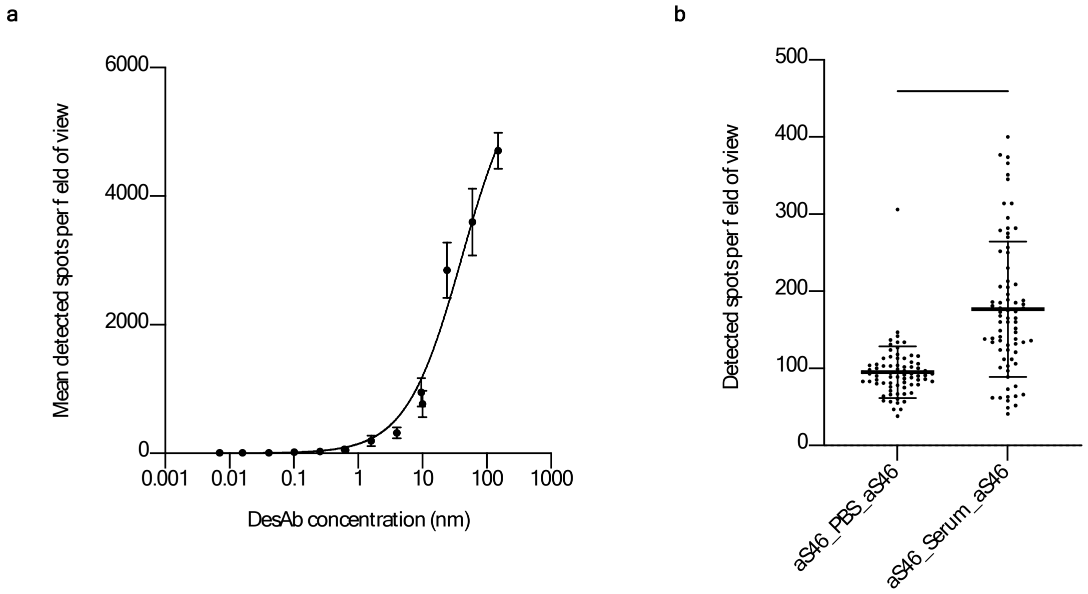

Figure S11. SiMPull experiments to estimate the binding affinity and the capturing ability of aS46 for α-synuclein aggregates in PD serum. (a) By varying the concentration of fluorophore-labelled aS46 and fitting the determined spots per field of view, a K_d of 41 nM (R^2=0.96) to α-synuclein aggregates detected in PD serum was estimated. **(b)** Biotinylated aS46 was used as capture antibody and fluorescently labelled aS46 as detection antibody. Data were analyzed using an unpaired t-test. **** $p < 0.0001$.

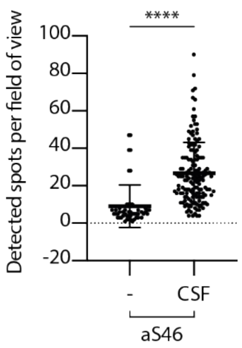

Figure S12. DesAb aS46 detects α-synuclein aggregates in the CSF of PD patients. The number of detected spots per field of view is shown. Error bars denote ± s.d. ($n \geq 50$). Data were analyzed using an unpaired t-test. **** $p < 0.0001$.

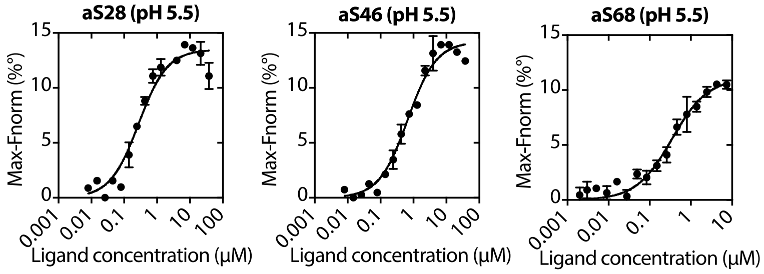

Figure S13. At low pH, aS28, aS46 and aS68 bind monomeric α-synuclein with low μM affinity. MST measurements showing the characteristic binding curve for three DesAbs at mild pH as used in kinetic aggregation assay. Error bars denote ± s.d. of three repeats.

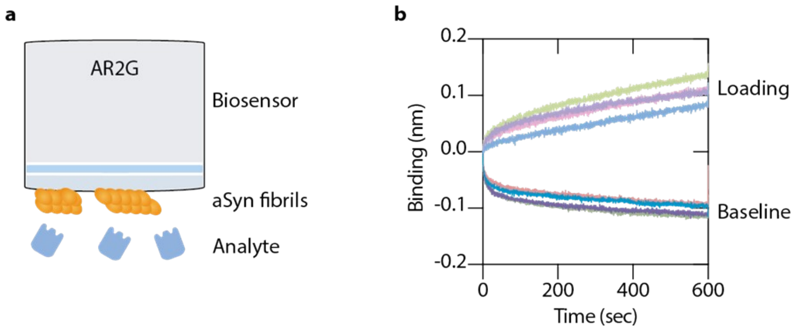

Figure S14. Experimental setup and sensor loading in BLI experiment. (a) Biosensor is loaded with α-synuclein fibrils. **(b)** BLI curves recorded for the loading of biosensors
